# Metabolomics in Children Cow’s Milk Protein Allergy: Possible Contribution from a System Biology Approach?

**DOI:** 10.3390/children11050562

**Published:** 2024-05-08

**Authors:** Alice Bosco, Veronica Altea, Paola Beretta, Roberto Cacace, Vassilios Fanos, Angelica Dessì

**Affiliations:** Department of Surgical Sciences, University of Cagliari and Neonatal Intensive Care Unit, AOU Cagliari, 09124 Cagliari, Italy; alice.bosco@unica.it (A.B.); v.altea@studenti.unica.it (V.A.); p.beretta@studenti.unica.it (P.B.); r.cacace@studenti.unica.it (R.C.); angelicadessi@unica.it (A.D.)

**Keywords:** metabolomics, CMA, system biology, food allergies

## Abstract

One of the most frequent triggers of food anaphylaxis in pediatric age but also among the most common, early, and complex causes of childhood food allergy is cow’s milk protein allergy (CMPA). The diagnostic course and management of this allergy is defined in a complex clinical picture due to several factors. First of all, the epidemiological data are not uniform, mainly as a consequence of the diagnostic methodology used in the various studies and the different age ranges covered. In addition, there is the complexity of terminology, since although CMPA traditionally refers to immune-mediated reactions to cow’s milk, it is a term encompassing numerous clinical features with different symptoms and the requirement for specific treatments. Moreover, the differential diagnosis with other very frequent diseases, especially in the first year of life, such as gastro-esophageal reflux disease or colic, is still complex. This can result in misdiagnosis and incorrect treatment, with harmful health consequences and significant economic repercussions. In this context, the combination of several omics sciences together, which have already proved useful in clarifying the allergenicity of cow’s milk proteins with greater precision, could improve the diagnostic tests currently in use through the identification of new, more specific, and precise biomarkers that make it possible to improve diagnostic accuracy and predict the patient’s response to the various available treatments for the recovery of tolerance.

## 1. Introduction

Food allergies (FAs) are a worldwide problem, affecting 6–8% of children, with the greatest impact in infants and very early childhood. They have been increasing over the past 20–30 years in developed countries, and this `appears to represent the evolution of the increase in the prevalence of allergic diseases that began during the second half of the 20th century [1].

In this context, cow’s milk protein allergy (CMA) represents not only one of the most frequent reasons of pediatric food anaphylaxis but is also among the most common, early, and complex causes of childhood FA. However, epidemiological data are not uniform due to the influence of many factors, especially the diagnostic criteria used in the various studies [2,3,4] and the different age ranges considered [5]. In fact, the British Society for Allergy and Clinical Immunology (BSACI) guidelines report a prevalence range for CMA between 1.8 and 7.5 percent in the first year of life [6], with data from parental reports or serum-specific IgE (sIgE) assessment showing higher rates of CMA than those from studies using an oral provocation test, considered the gold standard [2,3,7]. Confirming this are data from a systematic literature review on the prevalence of CMA in Europe, which showed an overall pooled estimate for all age groups of 6 percent, with prevalence diagnosed by food challenge of 0.6 percent [8]. The variation in prevalence is also correlated with the age groups considered, with ranges of 0.6–3% for children younger than 6 years, 0.3% in older children and adolescents, and less than 0.5% in adults [8,9]. In fact, the clinical manifestation of such allergy usually occurs within the first year of life and has a high probability of tolerance recovery by four years of age, with more delayed rates of resolution of IgE-mediated CMA than non-IgE-mediated CMA, although some patients still manifest reactivity in adulthood [3,10].

An additional complexity factor stems from terminology, as although CMA traditionally refers to immune-mediated reactions toward cow’s milk, it represents a term that encompasses numerous diagnostic facets with distinct symptoms, pathophysiology, and treatment [1].

This is compounded by the difficulty in differential diagnosis with other very common conditions, especially in the first year of life, such as gastro-esophageal reflux disease (GERD) or colic. In fact, it is still complex to discriminate between the various disorders because of the similarity in symptoms and the lack of practical and accurate diagnostic tests [11]. This can result in misdiagnosis and incorrect treatment, such as prescribing inappropriate medications or special formulated milks, with harmful health consequences and significant economic repercussions [3].

In this regard, an important contribution can come from the “omics” sciences, which have already proved useful in clarifying the allergenicity of cow’s milk proteins with greater precision, thus expanding knowledge about the pathogenesis itself [12]. Indeed, proteomics has been used to demonstrate a reduction in the final immunoreactivity of milk cooked within a matrix (wheat for muffin preparation) compared to heated milk alone (180 °C for 10 min) [9].

Additionally, a clearer differentiation of the several subtypes of CMA along with the prediction of the persistence of that allergy is now possible. However, the goals of the new technologies are much broader, and there is in fact the intent to overcome some of the limitations of currently used diagnostic tests to improve diagnostic accuracy and predict patient response to different treatments. This is possible through the identification of new, more specific, and accurate biomarkers that allow the identification not only of an individual’s threshold doses but also of tolerance to dairy products that have undergone heat treatment [12]. The application of systems biology in the clinical practice of CMA would seem useful in order to allow the integration of the latest and most innovative data from the individual patient with his or her medical history in an effort to create a tailor-made treatment [12].

## 2. Cow’s Milk Allergenicity and CMA Classification

The high protein content of cow’s milk, 30–35 g per liter, is provided by a pool of more than 25 different proteins. The two main protein categories are caseins, (αS1-casein, αS2-casein, β-casein, and k-casein), about 80% of the total, and serum proteins (β-lactoglobulin, α-lactalbumin, bovine lactoferrin, bovine serum albumin, and bovine immunoglobulins), the remaining 20% [8]. The role of these proteins in the pathogenesis of CMA is not yet fully elucidated. Indeed, although more frequent binding of IgE to the most abundant milk proteins, namely caseins and β-lactoglobulin (BLG), has been observed, all milk proteins appear to be potential allergens, even those that are present in trace amounts such as lactoferrin. What is more, the prevalence of sensitization to different proteins has varied over the years, probably due to both new milk processing methods and improved analytical-diagnostic technologies [13]. In this regard, to date, it is clear that the stability of cow milk proteins (CMPs) is closely related to their allergenicity; in fact, both high temperatures and the interaction between CMPs and matrix components result in irreversible aggregation of proteins into complexes of various molecular sizes that can affect the allergic response by altering the affinity and binding stability of both IgE and IgG [8]. Furthermore, the term CMA, although generically encompassing adverse reactions to cow’s milk, defines in clinical practice several distinct conditions that require a specific approach. [1]. In fact, it can be further subclassified as immunoglobulin E (IgE)-mediated FA, non-IgE-mediated FA, or mixed IgE and non-IgE-mediated FA [1,2].

IgE-mediated allergy, about 60 percent of cases, is a type I hypersensitivity reaction in which symptoms usually occur within minutes to 1–2 h after ingestion. In detail, the reaction is caused by the initial binding of specific IgE to mast cells, which results in mast cell degranulation and the release of inflammatory mediators, including histamine. This mechanism is responsible for symptoms such as urticaria, angioedema, throat tightness, respiratory symptoms including difficulty breathing, coughing and wheezing, gastrointestinal symptoms including abdominal pain, vomiting and diarrhea, and finally cardiovascular symptoms including dizziness, confusion, and hypotension [2]. This type of CMA is more easily identified due to the relative earliness of symptom manifestation after ingestion of cow’s milk [3].

In contrast, mixed and non-IgE-mediated forms have different underlying mechanisms and clinical presentations that make them more complex to identify, which is why the Cow’s Milk related Symptom Score (CoMiSS^TM^) has also been developed [2,3]. It represents a clinical tool aimed at raising awareness of the presence, intensity, and monitoring of clinical manifestations potentially related to cow’s milk (CM) intake but without delineating itself as a stand-alone diagnostic tool for CMA [7]. The diagnostic complexity stems mainly from the longer time interval between cow’s milk ingestion and symptoms (from a couple of hours to a few days) and the symptomatic similarity to pediatric functional gastrointestinal disorders (FGIDs), including regurgitation, vomiting, diarrhea, and constipation [2,3,7].

Specifically, non-IgE-mediated forms of CMA include food protein-induced enteropathy (FPE), food protein-induced allergic proctocolitis syndrome (FPIAP), food protein-induced enterocolitis syndrome (FPIES), and Heiner syndrome [2,3]. In contrast, regarding mixed forms of CMA (IgE and non-IgE mediated), they include eosinophilic allergic esophagitis, eosinophilic gastritis, and atopic dermatitis (AD) [2].

## 3. Cow Milk Allergy Diagnosis and Management

To confirm or exclude the diagnosis of CMA, as well as to assess the tolerability of CM in a child with a previous FA or to identify the threshold of reactivity, an oral provocation test should be performed for both IgE-mediated and non-IgE-mediated reactions. In fact, the double-blind, placebo-controlled oral test (DBPCFC) is the diagnostic gold standard for CMA, although the unblinded oral test (OFC), although less rigorous, has proven to be a well-validated tool in very young children. Both cases involve oral administration of the suspected allergen in a controlled, standardized setting [2,14]. However, the longer time required, high cost, and inherent risk of anaphylactic reaction make the application of such methods more complex in both clinical practice and large epidemiological studies [2]. Therefore, oral provocation tests are frequently replaced by serologic assessment of allergen-specific IgE, sIgE, or skin tests, SPT [2]. These diagnostic strategies are characterized by good sensitivity but low specificity, often being responsible for positive results in nonallergic subjects who are thus unnecessarily placed on an exclusion diet [11]. We also observe, in the case of, for example, FPIES, negativity of both skin and blood tests and much delayed symptomatology, such as severe vomiting at least 2 h after CM ingestion, complicating the diagnostic process [2]. Thus, it seems clear that the application of these tests essentially allows a prediction regarding the likelihood of developing an allergic reaction but is not sufficient to make a diagnosis, and their use may be a possible cause of incorrect prevalence estimates of CMA [2,3,7]. Nevertheless, patients with higher levels of specific sIgE and STP wheel size have been shown to have a higher probability of reaction during an oral provocation test [15]. This prompted the search for a cut-off, both for sIgE and SPT wheel size, that could predict by itself whether a patient would react to an oral provocation test. However, the use of cut-offs in clinical practice must be carefully assessed according to the context. There are in fact two types of cut-offs, depending on whether they are based on a high positive predictive value (95% PPV) or a high specificity (95% specificity). In the first case, it is important to consider that these values are related to the prevalence of an allergy in the population being studied and therefore only applicable in allergy centers where a prevalence similar to that provided by scientific studies on the subject is assumed. Conversely, being able to use cut-offs based on high specificity makes it easier to select cases for OFC testing [15]. In this regard, a systematic literature review of studies that analyzed the PPV of sIgE and SPT wheel size in the diagnosis of allergy to fresh and cooked CM according to age in order to identify possible cut-offs useful in clinical practice confirmed the presence of different results precisely according to the age analyzed. Indeed, it was shown that none of the cut-offs proposed in the literature can be used to definitively confirm the diagnosis of allergy to cow’s milk, neither fresh pasteurized nor cooked. However, it was found that in children under 2 years of age, the cut-offs for sIgE or SPT wheel size seem to be more homogeneous, providing some perspective for their future use [11]. In fact, the BSACI guidelines already suggested not recommending oral provocation testing in children younger than 2 years of age with SPT wheel size ≥ 6 mm, as this value provided 100% specificity for an oral challenge [6].

This diagnostic complexity, especially given the absence of specific biomarkers, is confirmed by the Europrevall study, the only pan-European cohort study to have used the DBPCFC. Indeed, that research showed the prevalence of CMA in less than 1 percent of children up to 2 years of age, also pointing out that of all children with CMA, as many as 23.6 percent had no detectable specific antibodies against CM in their serum, especially those from Great Britain, Poland, the Netherlands, and Italy [16].

In fact, the very recent update of the World Allergy Organization’s (WAO) “diagnosis and rationale for action against cow’s milk allergy” (DRACMA) guidelines validates that if CMA is suspected, after a careful history and physical examination of the patient, dietary elimination of cow’s milk followed by its reintroduction should be carried out, confirming oral provocation testing as the standard diagnostic procedure for CMA after an elimination diet [3]. Indeed, it is remarked that both elimination and reintroduction of cow’s milk and its derivatives are essential not only to diagnose CMA but also to induce tolerance. Furthermore, the authors point out that although DBPCFC represents the gold standard and the best scientific approach for practical and economic reasons, in very young children, an OFC is recommended. It is also reiterated not to undertake an OFC in patients with a history of recent anaphylaxis and in FPIES unless there is uncertainty that CM is the causative food [3].

In IgE-mediated allergy, the diagnostic elimination diet requires a short time window (1–2 weeks), whereas in non-IgE-mediated CMA, approximately 2–4 weeks are required. Oral provocation tests for IgE-mediated CMA and more severe types of non-IgE-mediated CMA should always be undertaken under close medical supervision, while, for other forms of non-IgE-mediated CMA, reintroduction can be performed at home [3]. In addition, upon confirmed diagnosis, it is recommended that only testing of changes in sensitization status, i.e., an oral, supervised, or home provocation test, should be used to set the correct duration of dietary elimination. In fact, there is little scientific evidence regarding the duration of dietary therapy, and current guidance is based on the observation that many infants with CMA become tolerant between 9 and 12 months of age. Therefore, a therapeutic elimination diet is usually recommended for at least 6 months, or until the age of 9–12 months, and if reintroduction causes symptoms, to continue the elimination diet for another 3–6 months and then reintroduce CM again. The authors also recommend that an OFC should not be substituted for the multi-step milk scale, even for diagnostic confirmation. Nonetheless, they endorse its use for reintroduction in non-IgE-mediated FA (FPIAP, FPE) and envisage its possible use in carefully selected cases of IgE-mediated CMA and CM-FPIES for tolerance assessment after a period of therapeutic elimination diet. Indeed, with the multi-step milk ladder, an initial replenishment with cooked milk is initiated, followed by a slow and gradual transition to less heat-processed milk. However, one may see tolerance to cooked milk and persistence of sensitization to unprocessed milk, so standardization of foods included in the ladder and careful and thorough medical care is essential. Instead, for prevention of anaphylaxis and severe response to accidental exposure, in patients with severe and persistent IgE-mediated CMA, oral immunotherapy (OIT) may be undertaken in specialized centers. It consists of daily ingestion of increasing doses of allergen during the up-dosing phase and intake of a constant dose during the maintenance phase, based on specific individualized protocols [3].

In mixed forms of CMA (IgE and non-IgE mediated), identification of cow’s milk as a trigger should be performed through careful history taking and diagnostic elimination diet when necessary [2,3]. In fact, in the case of AD, FA is only one of the possible triggers in about one-third of patients, and the most recent indications suggest that in such individuals, only after establishing optimal skin treatment is it appropriate to proceed with allergy testing. Where this proves positive, together with a clinical reactivity clearly attributed to the food, one can proceed with an elimination diet that is well tailored and appropriate to improve the severity of AD itself [17,18].

## 4. Origin of Cow Milk Allergy

Already during fetal life, the infant’s immune system begins to shape itself according to the external environment. This condition is responsible for potential allergic sensitization of the fetus, particularly as a consequence of swallowing that generates intestinal priming of the immune response, through lymphoid accumulations in the small intestine, especially in an atopic maternal environment. However, there are some factors that counterbalance this imbalance in the immune response, the most important of these being fetal production of interferon gamma INF-γ, maternal supply of the soluble protein CD14, and maternal G-type immunoglobulin (IgG) [19]. Therefore, it has been hypothesized that high maternal cow’s milk intake could increase milk-complexed IgG antibodies by reducing the risk of CMA in the unborn child [1].

After birth, additional factors contribute to the full maturation of the child’s immune system, but a healthy microbiota certainly represents the most significant. It diversifies in the first years of life, reaching a condition of eubiosis similar to an adult around the age of three years, and in this process, there is an important contribution of both breast milk, due to the presence of unique prebiotics, the oligosaccharides (HMOs), and proper nutrition starting from weaning [1].

Over the years, the role of nutrition, especially weaning, in the context of allergic diseases has been extensively studied, contributing to new guidelines for health professionals [17,20]. In fact, although increased IgE-mediated reactivity to food proteins has been found in many patients with allergic diseases such as AD [17,21,22,23,24,25], according to some authors, their relationship should be interpreted in a multimorbidity framework, thus their co-occurrence does not imply any specific relationship between them and certainly not a progressive or causal relationship, thus questioning the legitimacy of the atopic march model [1,17,21,22,23,24,25]. Indeed, while, traditionally, the allergic march has been described as a sequence beginning with FA, it is nowadays known that allergic diseases are complex, multifactorial, and caused by a variety of distinct mechanisms responsible for multiple heterogeneous clinical phenotypes [1,21,22,23,24,25]. However, some authors argue for the existence of some mechanistic explanations for the progression of atopic march in a proportion of affected individuals, specifically those with an altered skin barrier [1,22,23,24,25]. In fact, in AD, the genetic component of the disease, by affecting the polymorphism in the gene coding for filaggrin, is responsible for a dysfunctional skin barrier [1,26]. This causes a high skin permeability that results in the possibility of increased penetration of both food allergens and inhalants into the dermis, leading to sensitization. However, only one-third of patients with AD have FA. In this regard, a multiomics study conducted by Leung et al. [27] showed that the more superficial compartment of uninjured skin in atopic subjects with FA has distinctive features compared with other atopic endotypes.

These considerations suggest the possibility of reversing the order of causation traditionally recognized by the atopic march, placing FA as a consequence of the gene defect contributing to AD and not its cause [1,28]. In fact, according to the results of a population-based study by Martin et al. [29], the likelihood of a child developing FA is closely related to the earliness of onset and severity of AD. In detail, a population of children with AD underwent a four-food SPT (egg white, peanut, and sesame, as well as cow’s milk or crustacean) and then were subjected to further follow-up where, in addition to repeating the SPT, serum allergen-specific IgE was measured and oral food challenges to peanut, egg white, or sesame were performed. Unfortunately, it was not possible to subject them to OFC to cow’s milk due to the scarcity of resources. This made it possible to classify the children as sensitized to egg white, peanut, cow’s milk, or sesame on the basis of SPT or the level of food-specific IgE in the serum, and further categorization as allergic or tolerant to egg white, peanut, or sesame followed the OFC. The results showed in children with AD not only a higher risk of specific food allergies to egg white, peanut, or sesame but also a higher risk of sensitization to egg white, peanut, milk, or sesame [28]. In this regard, the strong correlation between skin barrier disruption and oral tolerance has been well highlighted by studies on early egg introduction [30]. In fact, it was found that the synergy between early allergen introduction and AD treatment is the key to primary prevention of food allergy, also highlighting the insufficiency of only one of the treatments. It would thus seem that the counter-regulatory mechanisms of early oral exposure in AD patients are important in maintaining tolerance. Nevertheless, according to a very recent systematic review of the literature, although early introduction of more allergenic foods in the first year of life has been associated with a lower risk of developing food allergy, the level of evidence regarding the timing of the introduction of cow’s milk and the risk of milk allergy is low, and further studies are also needed to develop interventions on allergenic foods that are safe and acceptable to children and their families given the high dropout rate [31].

On this matter, skin sensitization to cow’s milk is most likely given its ubiquity in home environments [1].

## 5. Cow Milk Allergy Primary and Secondary Prevention

The literature’s data show that rates of spontaneous resolution of CMA are slowing [32]. Therefore, it appears necessary to understand the pathogenetic mechanisms and develop preventive strategies for FA, especially in the presence of family history of allergy, still considered the most important risk factor for offspring. Indeed, the application of primary preventive strategies to decrease the onset of the disease, at least in high-risk children, i.e., those with a first-degree relative with an allergic history, would be useful. In addition to this, there is a further possibility of intervention, through secondary prevention, aimed at preventing the progression of the disease, from mild-to-moderate to severe, through the recovery of tolerance [1].

The main modifiable factors for the application of primary preventive strategies are as follows: maternal diet in pregnancy and lactation, breastfeeding, use of special formulated milks, weaning, topical emollients, and probiotic supplementation [1]. Regarding manipulation of the maternal diet during pregnancy and lactation, there are few data to support its usefulness [33,34], except in very rare cases, as reported in the latest guidelines for the management of FA by the Global Allergy and Asthma European Network (GA2LEN), a multidisciplinary international task force [35]. Indeed, it is specified not only that breast milk remains the preferred nutritional option in cases of CMA, as reported by other guidelines [36], but also that infants with IgE-mediated CMA are rarely so sensitive as to react to the very low levels of food allergens in breast milk, thus reiterating that the harm resulting from nutritional inadequacy during breastfeeding may outweigh any benefit for the management of FA in infants [35]. It is therefore recommended that lactating mothers with CMA infants consider an elimination diet only on the specific, strictly individual advice of the health care professional after a careful medical history. These recent recommendations seem to outweigh the findings of some studies that cow’s milk avoidance in nursing women has been shown to be associated with lower casein and BLG IgA levels and the development of CMA in infants [37].

On the other hand, with regard to pre- and/or probiotic supplementation, both to the pregnant/lactating mother and in the early years of the child’s life, further studies are needed to assess their real preventive efficacy because although there are data regarding their usefulness in preventing the onset of Th2-mediated allergic diseases, final evidence cannot yet be provided [38].

A very recent literature review that addressed the potential prophylactic effects of breastfeeding on FA made it clear that breast milk is definitely effective in providing partial protection to infants [39]. In fact, breast milk, especially colostrum, given its content in active immune factors, such as antibodies, cytokines, inflammatory mediators, signaling molecules, and soluble receptors, can reduce the risk of allergic diseases and promote tolerance [1,39]. There is also the contribution of HMOs, especially for their probiotic action, given the key role of the gut microbiota in tolerance induction [1]. Indeed, a recent systematic review of the literature found that low concentrations of lacto-N-fucopentaose III (LNFP-III) would appear to be associated with the onset of CMA [40]. However, the relationship between the duration of breastfeeding and the incidence of FA in early childhood remains unclear, although the contribution of prolonged maternal breastfeeding seems to be evidenced not only in promoting tolerance during complementary feeding but also in counteracting the early introduction of solid foods [39,41,42].

In this regard, the importance of proper timing of complementary feeding’s initiation, certainly after 17 weeks, preferably around 6 months of age, and exposure of the child to all potentially allergenic foods, without delayed introductions of allergens, is recognized to date to promote the acquisition of tolerance precisely through early oral exposure [20,30,39,41,42]. The importance of this mechanism and the difference from skin sensitization was proposed by recent work on the correlation between AD and FA conducted by Eigemann et al. [17]. In fact, normally, tolerance induction occurs through the conversion of naïve T cells into regulatory T cells (Treg), which are responsible for the inhibition of IgE development and thus FA through the action of CD103+ dendritic cells, triggered by the production of mucin by intestinal epithelial cells and granulocyte-macrophage colony-stimulating factor (GM-CSF) by innate lymphoid cell type 3 (ILC3). In contrast, in the presence of an altered skin barrier, the increased permeability of allergens, especially those ubiquitous in the home environment such as eggs and cow’s milk, results in the activation of the Th-2-mediated immune response by ILC2, dendritic cells, and basophils, which are responsible for the production of specific IgE [17]. These findings support the already discussed need to subvert the order of causality traditionally recognized by the atopic march, and in this light lies the possibility of prevention through the application of topical emollients in order to promote proper skin barrier function and thus reduce sensitization by this route. However, the evidence for this is still debated [1,43,44,45,46].

The importance of nutrition in the earliest ages of development also with regard to the prevention of FA emerges clearly from a very recent work published by Paparo et al. [47]. Indeed, they conducted the first work investigating the role of some potentially harmful components of ultra-processed foods (UPFs), the advanced glycation end products (AGEs), byproducts of the Maillard reaction, in the onset of FA. In fact, evidence is now conspicuous regarding the possible correlation between UPFs, ready-to-eat or ready-to-heat industrial formulations of processed food substances (oils, fats, sugars, starch, protein isolates) subjected to hydrolysis, hydrogenation with the addition of flavorings, coloring agents, emulsifiers, and other additives and the onset of chronic noncommunicable diseases [47]. The objective of this investigation, conducted through different experimental models (human enterocytes, human small intestine organ cultures, and peripheral blood mononuclear cells from children at risk of allergy) was to evaluate the effects of the three most common glycation products in foods in the Western diet, Nε-(carboxymethyl) lysine, Nε-(1-carboxyethyl) lysine, and Nd-(5-hydro-5-methyl-4-imidazolone-2-yl)-ornithine (MG-H1), in children with FA and healthy age-matched controls. The results showed that human enterocytes exposed to AGEs exhibit alterations in intestinal barrier, AGE receptor expression, reactive oxygen species production and autophagy, with increased transepithelial passage of food antigens, responsible for possible negative impact on immune tolerance [47].

**Figure 1:**
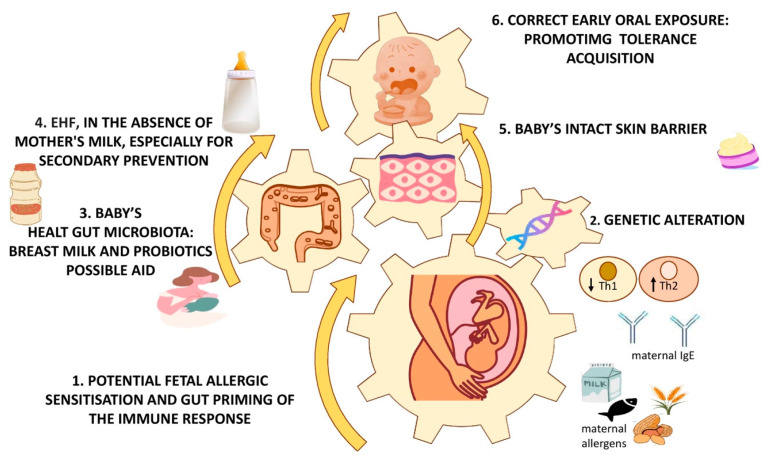
Main factors influencing the CMA development and possible targets for preventive interventions. Abbreviations: ↓, low; ↑, high; eHF, extensively hydrolyzed formula; Th1-2, Th1-2 lymphocytes; IgE, Immunoglobulin E.

Finally, the preventive contribution of partially hydrolyzed formulas (containing peptides with molecular weights less than 5000 Da) in a primary prevention perspective has always been debated, certainly also due to the absence of consensus regarding the correlation between early exposure (first weeks of life) to CMPs and the risk of CMA later in life [1]. In this regard, the latest Cochrane review [48], which compared the effects on allergic disease in infants fed a hydrolyzed formula versus classical formula (CMF) or human breast milk, confirmed the absence of evidence to support the preventive role of feeding, both short-term and prolonged, a hydrolyzed formula versus exclusive breastfeeding. They also pointed to the absence of evidence that prolonged feeding with a hydrolyzed formula compared with a CMF is useful for the prevention of allergic disease in infants who cannot be exclusively breastfed, although there is very low-quality evidence regarding the short-term use of an EHF compared with a CMF in the prevention of infant CMA [48]. However, with regard to secondary prevention, data in the literature regarding the use of particular formulas to hinder the progression of CMA provide stronger evidence in the case of non-breastfed infants. In fact, the first choice for formula-fed infants with CMA is the use of particular extensively hydrolyzed (eHFs) formulas containing small peptides from cow’s milk, despite the presence of significant differences in the molecular weights and peptide profiles of eHF found on the market. The use of this strategy is definitely of first choice to promote a recovery of tolerance in patients with moderate CMA, while, in more severe cases, such as in case of failure of treatment with eHF or in the presence of infants with very severe symptoms, such as anaphylaxis or multiple food intolerances, amino acid-based formula (AAF) should be opted for. This formula, composed of free amino acids, although free of antigens, would indeed appear to be unsuitable for tolerance recovery. There is still the possibility of using only partially hydrolyzed formulas (pHFs) in mild cases of CMA or during complementary feeding, prior to the introduction of unmodified cow’s milk, as they are potentially considered safer than the use of cooked milk [1]. In addition, evidence is increasing regarding a positive effect on the acquisition of immune tolerance in patients with CMA treated with eHF supplemented with specific probiotics, such as Lactobacillus GG [49,50,51], or specific HMOs, such as 2′-fucosyl-lactose and lacto-N-neotetraose [52], supporting modulation of the gut microbiota as a possible target for secondary prevention intervention. Figure 1 summarizes the main factors that may influence the development of CMA from the fetal period until weaning, also offering useful targets for preventive purposes.

## 6. Metabolomics and Systems Biology Approach to CMA

The global increase in FA is not stopping despite advances in both preventive and therapeutic strategies [3,47]. There are several reasons for this; on the one hand, the application of therapeutic strategies such as OIT is often limited by the need for continuous long-term exposure to the allergen and, on the other hand, effective prevention campaigns require more precise identification of target populations [53]. Indeed, although the development of FA often appears to be related to other atopic diseases (atopic march), such as AD, there is wide variability in the clinical development of FA, supporting the presence of different endotypes (specific pathogenetic pathways) of disease. They are likely the result of dietary, environmental, genetic, epigenetic, and psychosocial factors that determine specific phenotypes of allergic individuals; however, the molecular mechanisms responsible for this clinical heterogeneity have not yet been identified, making it difficult nowadays to predict the persistence of allergy or the possibility of transient desensitization rather than sustained unresponsiveness (SU, desensitization of more than 1 year that does not require continuous low-dose consumption of the allergenic food) [53,54,55,56,57,58]. Therefore, an innovative approach that can accurately characterize the molecular changes that occur both during the development of FA and in response to therapy is essential. This goal is certainly achievable through the application of modern omics technologies, which have already proven useful in identifying potential biomarkers in FA [53,54,59,60,61,62,63].

In this regard, metabolomics, one of the latest “omics” sciences, starting from the analysis of the complete set of metabolites (metabolome, generally molecules of molecular weight < 1500 Da) present in a given biological system allows us to photograph the genome in its interaction with the environment, thus providing detailed information on the metabolic status of an organism as a consequence of environmental influences, diet, lifestyle, and possible therapeutic strategies [64]. Indeed, metabolomics, by detecting broad classes of metabolites (including sugars, lipids, small peptides, vitamins, and amino acids) present in cells, tissues, organs, and biological fluids allows the detection of a complete functional phenotype encompassing both clinical features and genetic and nongenetic factors [65].

Nonetheless, the onset of FA risk early in development and the heterogeneity of molecular mechanisms responsible for the clinical variability of FA require large-scale cohort studies in an attempt to create early profiling suitable for the identification of predictive biomarkers that can allow risk stratification according to different allergic phenotypes [47]. In this regard, the systems biology approach, in an attempt to understand the whole system rather than individual aspects of it, through the integrated use of high-throughput analytical technologies and machine learning, may be the key to the development of a much stronger predictive model than any single approach [53,66]. Therefore, a holistic approach to more clearly define the phenotypic heterogeneity of FA is essential, and through bioinformatic interpretation of all the data from the different omics, from DNA sequencing to global transcriptional profiling to individual metabolites, it will be possible to understand the problems of a complex biological system such as the human being [67].

Nevertheless, to date, most studies regarding CMA have been based on a single omics approach; in fact, only a few studies have provided an integrated analysis. Table 1 shows all metabolomics studies on CMA conducted to date, some of which have included an integrated approach with analysis of the gut microbiota using innovative culture-independent techniques (microbiomics). Such a synergy of investigation appears crucial given the important role of the microbiota in mediating the antigenic response by modulating the balance between Th1 and Th2 lymphocytes [68].

The first pioneering studies in this regard were conducted by Thompson-Chagoyan et al. [69] in 2011 and by Berni-Canani et al. [70] in 2015. The first group compared the fecal microbiota, analyzed by fluorescent in situ hybridization and flow cytometry, and the production of acetic, propionic, butyric, isocaproic, and branched-chain short fatty acids (BCSFAs), as measured by gas chromatography, in children with CMA compared with healthy controls between 2 and 12 months of age. The results revealed the presence of significantly higher proportions of the *Clostridium coccoides* group, the Atopobium cluster, and a greater sum of the proportions of the different bacterial groups in children with CMA. This supports the importance of gut dysbiosis in the pathogenesis of this allergy, although no decisive role emerged for any single species or genus. In contrast, analysis of selected metabolites revealed higher concentrations and percentages of butyric acid and BCSFA in the feces of infants with CMA despite the fact that both fecal pH and ammonia were unchanged between the two groups [69].

In contrast, Berni-Canani et al. [70] studied the effect of supplementation with *Lactobacillus rhamnosus GG* (LGG) of extensively hydrolyzed casein-based formulas (EHCF). Fecal samples from 19 infants with CMA, including 12 treated with EHCF + LGG and 7 with EHCF alone, before and after treatment, were compared with 20 healthy controls. Again, a CMA-related dysbiosis emerged, with Lachnospiraceae and Ruminococcaceae dominating in the case of CMA compared with eubiosis characterized by the prevalence of Bidobacteriaceae and Enterobacteriaceae. In terms of treatment efficacy, however, *Blautia*, *Roseburia*, and *Coprococcus* increased significantly after treatment with EHCF + LGG, but only the genus *Oscillospira* was significantly different between children who became tolerant and those who remained allergic. Increased fecal butyrate levels, measured by gas chromatography, also characterized most of the children who achieved tolerance. In addition, analysis of individual bacterial strains showed specific demarcations between tolerant and allergic children even in the taxa *Blautia* and *Roseburia*. These findings prompted the authors to hypothesize that treatment with EHCF + LGG promotes tolerance in infants with CMA in part by influencing the bacterial community structure at the strain level of the infant’s gut.

A few years later, Seppo et al. [71] continued to search for evidence to support the role of the infant’s gut microbiome in CMA by investigating, through a metabolomics analysis conducted with HPLC, the oligosaccharide composition of breast milk (HMOs) received by infants who developed CMA compared with non-CMA infants. The results of that analysis showed that after adjustment for infant age and maternal covariates (including atopic disease, duration of breastfeeding, and secretor status), the infants found to have CMA had consumed milk containing lower levels of 6′-sialyllactose (6′SL), disialyllacto-N-tetraose (DSLNT), lacto-N-fucopentaose I and III (LNFP I and LNFP III), and a tendency to also have lower levels of LS-tetrasaccharide c (LSTc). However, following correction for multiple comparisons, only the level of LNFP III remained significantly lower in the milks of mothers with a CMA infant even though 6′SL, LSTc, and LNFP III (group a) formed a co-expressed cluster, which, together, was found to be significantly correlated with CMA status. In addition, further classification according to the type of CMA showed that in all cases of delayed-onset CMA, the milk contained 2′-Fucosyllactose (2′-FL) and LNFP I (mothers with secretory phenotype) in contrast to mothers with an infant with immediate-type (IgE-mediated) CMA. In light of what they observed, the authors hypothesized that it would be the Lewis X antigen, present in LNFP III, and not FUT2 (secretor status) that would be associated with protection against CMA even if the co-expressed cluster of 6′SL, LSTc, and LNFP III does not have a common biosynthetic pathway, making it complex to understand what regulates this expression pattern. Indeed, they concluded that the presence of other molecules in breast milk that may have influenced the development of CMA cannot be ruled out and that higher concentrations of LNFP III, although shown to be associated with the failure to develop CMA, are not necessary to prevent such allergy, involving other potential mechanisms [54].

In contrast, Adel-Patient et al. [72] searched for the existence of a metabolomic signature characteristic of CM-FPIES. They compared children with CM-FPIES and control subjects with IgE-CMA, both of whom were on exclusion diets. Untargeted metabolomic analysis of plasma collected before the oral provocation test was conducted by two complementary LC-MS methods, which were then analyzed by univariate analysis. The obtained plasma metabolic profiles allowed identification of a specific CM-FPIES metabolomic signature. Indeed, metabolites were observed significantly discriminating between CM-FPIES subjects and both active and resolved IgE-CMA subjects (three children initially recruited for IgE-CMA had negative OFC). Specifically, CM-FPIES subjects were found to have significantly lower concentrations of alpha-hydrostearic acid, 2-hydroxyicaproic acid, myristic acid, palmitic acid, and other unidentified saturated and methyl fatty acids. In contrast, the levels of some amino acids and their derivatives were higher in CM-FPIES than in IgE-CMA patients but less clearly than in IgE-resolved patients. The concomitant analysis of humoral and cellular immune responses showed lower concentrations of total Ig in CM-FPIES children than in control subjects, with specific IgE against CM components weak to undetectable and the complete absence of specific IgE against CM digestion products. Children with CM-FPIES were also found to be characterized by the absence of both Th cell proliferation and associated cytokine secretion following allergen reactivation, all features found in children with IgE-CMA [72].

Of very recent publication is the work of Shibata et al. [73], an ancillary cohort study of a randomized multicenter trial of children with IgE-mediated CMA who underwent OIT for 13 months. The aim of the work was to research changes in gut environmental factors and their association with SU acquisition in OIT for school-age children with IgE-mediated CMA. SU in the study was defined as the ability to consume cow’s milk above the target dose in a double-blind, placebo-controlled dietary challenge after OIT followed by a two-week dairy elimination diet. A longitudinal collection of 175 fecal samples was conducted followed by clustering of microbiome and metabolome data into 29 modules inherent to microbial populations and 12 modules for water-soluble fecal metabolites (WSM). The results showed that although OIT improved immunological parameters, the probability of SU acquisition was low (only 7 SU in 28 children). This multiomics approach also made it possible to analyze both changes in the gut microbiota and WSMs during treatment. Regarding gut environmental factors, substantial changes during OIT were limited to the early periods of therapy; in fact, relevant changes in some fatty acids and their conjugates differed significantly at the beginning of OIT, while they returned closer to baseline levels at the end of treatment. In contrast, analysis of the microbiota found that higher eigenvalues of a Bifidobacterium-dominant module (Mb-24:Bifidobacterium) were associated with a higher likelihood of obtaining SU. Lower levels of milk- and casein-specific IgE also correlated positively with tolerance acquisition. In addition, a correlation was found between these three SU-associated factors (higher eigenvalue of a Bifidobacterium-dominant module, lower levels of milk- and casein-specific IgE) and other gut environmental modules, especially a module of the gut microbiota Mb-09: Lachnospiraceae and WSM being part of the monosaccharide group. These two modules contain many components that potentially act in gut protection and sugar metabolism; in fact, they are positively associated with immunity and mucosal integrity, supporting their importance in oral tolerance to food allergens [73].

Further evidence for the usefulness of the multiomics approach can be seen in the work of Boulangé et al. [74]. With the aim of studying the effects on the microbiota and fecal metabolome of an extensively hydrolyzed whey-based formula (EHF) supplemented with two breast milk oligosaccharides, 2′-FL and lacto-N-neotetraose (LNnT), they conducted a randomized, multicenter intervention study of 194 infants (2 weeks to 6 months of age) with CMA, who were not breastfed, up to 12 months of age. The effects on the microbiota were studied by calculating phylogenetic alpha-diversity through a classification-based analytical approach that produced five clusters of samples, i.e., fecal community types (FCTs), characterized by similar taxonomic groups at the gender level. The results showed that supplementation of a whey-based EHF with 2′-FL and LNnT enriched the microbiome with HMO-utilizing Bifidobacteria and slowed the progression of microbiome composition toward an adult-type pattern rich in Firmicutes. Indeed, both reduced microbial diversity and enrichment of FCT early stages were observed in the HMOs group. It was also found that such supplementation partially reversed the dysbiosis characteristic of CMA infants, making the microbiome more similar to that normally present in breastfed infants, rich in *B. longum* subsp. *Infantis*, *B. breve*, *B. bifidum*, and *B. longum subsp*. *longum.* With regard to the fecal metabolome, specific HMO-mediated changes in SCFAs (acetic acid) production, fecal amino acids degradation, and bile acids conjugation were observed, particularly in infants who started formula supplemented with HMOs before three months of age. In contrast, in the group of infants enrolled at an older age who were already exposed to solid foods, the changes in the gut microbiome mediated by HMO were less pronounced, with significant differences detected only at 12 months of age. This led the authors to hypothesize that at later life ages, a longer intervention may be needed to induce an effect [74].

The objectives and possible contribution from the application of systems biology and omics technologies to CMA are summarized in Table 2, along with the possible clinical findings and the studies that have addressed them.

Other omics technologies have been used to elucidate other aspects of CMA, however, without an integrated approach. Thanks to proteomics studies, it has been possible not only to characterize milk allergens more precisely and identify new ones but also to detect the presence of traces of allergens in complex food matrices, even after damage to their chemical or physical structure, unlike with immunochemical methods. These findings provide the basis for the design of new diagnostic tests that are more sensitive and specific than traditional methods while also clarifying the presence of acute signs and symptoms of CMA in patients without high levels of sIgE against proteins assessed by most tests in use [12,75]. To this should be added the potential applications of proteomics in the treatment of CMA, not only by monitoring the processing of cow’s milk hydrolysates but also to improve OIT through hypoallergenic molecules containing T-cell epitopes but lacking IgE epitopes, increasing the safety of methodic take. Finally, clinical proteomics can enable monitoring of a therapy by specific circulating biomarkers [75].

**Figure 2:**
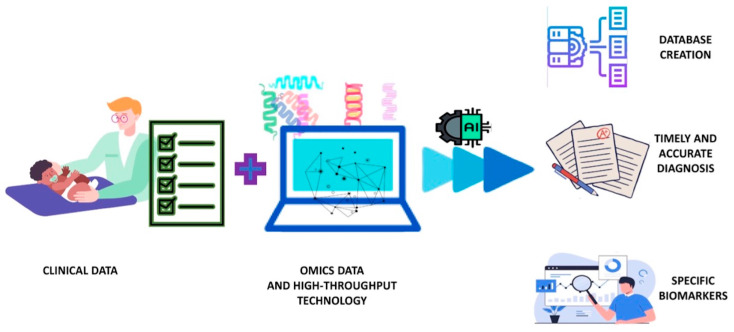
Application of the systems biology approach to the clinic in CMA.

The new approach and the related contribution from applying the systems biology approach to clinical settings in the study of CMA are schematized in Figure 2.

Finally, in clinical practice, molecular-based allergy diagnostics (MAD), capable of defining a patient’s allergen sensitization at the molecular level through the use of purified, native, or recombinant allergens, has begun to expand. In addition, it is possible to measure sIgE antibodies against multiple allergens in a single test in which the allergens are immobilized in a microarray using a small amount of serum, thus identifying a patient-specific immunoreactive profile, which is useful both in attempting to define disease severity and in providing information on the likelihood of overcoming allergy [75].

## 7. Conclusions

The extreme heterogeneity of the pathogenetic pathways of CMA results in different disease endotypes that need to be correlated more precisely with clinically relevant allergic phenotypes. This variability, to date, is weakly reflected in the most common treatment guidelines, requiring an innovative approach based on precision medicine [58].

This could be achieved through the integration of different omics, which, thanks to high-throughput technologies, are capable of generating large-scale, big data, information collection whose size and complexity exceed the capabilities of traditional data processing applications [76]. The ways in which this complex set of information from the various omics can be analyzed, integrated, and utilized are possible through artificial intelligence, specifically machine learning, which can unravel the intricate workings of systems biology using predictive algorithms. This could allow the creation of large archives of multiomics data that precisely, through specific algorithms, in turn, can optimize diagnosis and predict therapeutic response [77]. It will then be possible to characterize allergic endotypes, understand allergic multimorbidity relationships, and contextualize the impact of environmental exposures (the exposome) and genetic risks [62].

Therefore, only through a comprehensive, systems biology-oriented approach will it be possible to identify and validate specific biomarkers useful not only in providing information on disease severity and future progression but also in therapeutic response. This will make it possible both to reduce the number of OITs with their associated health and economic side effects by optimizing diagnosis [75] and to validate specific biomarkers associated with safe and effective OIT, excluding individuals in whom OIT might pose unnecessary risks [78,79,80]. This makes possible the integration of multiple layers of data, specific to the individual patient, in order to optimize diagnosis and tailor treatments, with a goal of maximizing therapeutic efficacy and minimizing morbidity, mortality, and costs, guiding physicians in the management of patients with a view to increasingly personalized medicine [62,79,80,81]. Nevertheless, although this approach is promising, further studies are needed to assess its usefulness in everyday clinical practice.

## Figures and Tables

**Table 1:** CMA metabolomics studies.

Authors/Years	Omics Technologies	Samples	Bio-Specimens	Technique	Results	Clinical Significance
Thompson-Chagoyan et al. [69] 2011	Metabolomic and microbiomic	46 CMA infants and 46 healthy controls aged 2–12 months	Fecal samples	GLC Fluorescent in situ hybridization and flow cytometry, using a panel of 10 rRNA targeted group- and species-specific oligonucleotide probes	↑ proportions of the Clostridium coccoides Group, Atopobium cluster and sum of the proportions of the different bacterial groups in CMA infants ↑ concentrations and percentages of butyric acid and BCSFA in CMA infants	Correlation between gut dysbiosis and CMA although no single species or genus appears to play an essential role Bacterial fermentation’s products could represent biomarkers of the pathology
Berni-Canani et al. [70] 2015	Metabolomic and microbiomic	19 CMA infants before and 20 healthy controls after treatment with EHCF with (n = 12) and without (n = 7) supplementation with LGG	Fecal samples	GC 16s RNA	Blautia, Roseburia, and Coprococcus were significantly enriched by EHCF and LGG treatment only one genus, Oscillospira, discriminated between infants that became tolerant and those that remained allergic ↑ in fecal butyrate levels in most tolerant infants Blautia and Roseburia exhibited specific strain-level demarcations between tolerant and allergic infants	EHCF + LGG promotes tolerance in infants with CMA, influencing the strain-level bacterial community structure of the infant gut
Seppo et al. [71] 2017	Metabolomic	41 mothers of non-CMA infants and 39 mothers of CMA infants	Stored breast milk	HPLC	↓ LNFP III in the mothers with a CMA infant mothers with a non-IgE CMA infant were secretors (2′-FL and LNFP I), those with IgE CMA were not 3 seemingly unrelated HMOs, 6′SL, LSTc, and LNFP III (group a), formed a co-expressed cluster, which, together, significantly correlated with CMA status	The Lewis X antigen (FUT 3) that is present in LNFP III and not the FUT2 is associated with protection against CMA ↑ LNFP III concentrations are not required to prevent CMA, other mechanisms must be involved
Adel-Paxtient et al. [72] 2019	Metabolomic	9 children with CM-FPIES (3 children initially recruited for IgE-CMA but who experienced negative OFC, IgE resolved) and 12 control subjects (6 IgE)	Plasma samples	LC/MS UHPLC LC/ESI-MS-MS	↓ concentrations of various fatty acids: alpha-hydrostearic acid, 2-hydroxycaproicacid, myristic acid, palmitic acid, and other unidentified methyl and saturated fatty acids in CM-FPIES infant ↑ concentrations of some amino acids and their derivatives, purine metabolites, or vitamins in CM-FPIES patients vs. IgE-CMA patients but less clearly compared to IgE-resolved ones	Specific metabolomic signature identification for patients with CM-FPIES
Shibata et al. [73] 2023	Metabolomic and microbiomic	32 school-age children with IgE-mediated CMA who underwent OIT for 13 months	Fecal specimens	MS 16s RNA	↓ levels of milk and casein-specific IgE and ↑ eigenvalue of Bifidobacterium-dominant module are SU-associated factors and they correlated with other gut environmental modules, especially with Mb-09: Lachnospiraceae and WSM-04: Monosaccharides	Identification of clinical and gut environmental factors associated with SU acquisition in CM-OIT
Boulangé et al. [74] 2023	Metabolomic and microbiomic	190 non-breastfed infants with CMPA until 12 months of age randomized to receive either the HMOs-supplemented formula (*n* = 94) or control formula (*n* = 96)	Fecal specimens	UPLC-MS/M shotgun analysis	HMO intake ↓ the developmental progression toward a mature, adult-type microbiome composition ↑ Bifidobacteria and ↓ Proteobacteria after 1 and 3 months of HMO formula feeding ↓ fecal metabolites derived from the bacterial oxidative catabolism (Ehrlich pathway) of BCAAs and aromatic amino acids (isobutyric acid, isovaleric acid, phenylacetic acid, 3,4-hydroxyphenylacetic acid, and 4-cresol sulfate): ↓ of energy-forming amino acid catabolism bacterial bile acid deconjugation maintained at a stable level over time in the HMO group, while it ↓ in the control group, suggesting an HMO-mediated upregulation of BSH activity no significant differences in acetic acid concentrations between feeding groups, but HMO feeding for 1 month maintained ↑ fecal acetic acid levels compared to baseline levels, while it tended to ↓ in the control group.	Supplementation of a whey-based EHF with 2′-FL and LNnT partially corrected the dysbiosis commonly observed in CMA infants shifting the microbiome composition closer to a pattern typical of breastfed infants

Abbreviations: ↓, low; ↑, high; CMA, cow’s milk protein allergy; CM, cow milk; GLC, gas liquid chromatography; EHCF, extensively hydrolyzed casein formula; LGG, Lactobacillus rhamnosus GG; HPLC, high-performance liquid chromatography; 2′-FL, 2′-Fucosyllactose; LNnT, lacto-N-neotetraose; LNFP I/III, lacto-N-fucopentaose 1/3; FUT2, fucosyltransferases 2 secretor gene; FUT 3, Lewis X gene; IgE, Immunoglobulin E; CM-FPIES, cow milk food protein-induced enterocolitis syndrome; LC/MS, liquid chromatography–mass spectrometry; UHPLC, ultra-high-performance liquid chromatography; LC/ESI-MS-MS, liquid chromatography electrospray ionization tandem mass spectrometric; OIT, oral immunotherapy; WSM-04, water-soluble metabolite-04; SU, sustained unresponsiveness; BCAAs, branched-chain amino acids; BSH, bacterial bile salt hydrolase.

**Table 2:** Possible contribution from a systems biology approach to CMA.

Possible Contribution from a Systems Biology Approach to CMA		
Purpose	Clinical Relevance	Studies
Predictive biomarker research	Effective identification of allergy and reduction of side effects associated with DBPCFC or OFC	Thompson-Chagoyan et al. [66] 2011 Adel-Paxtient et al. [69] 2019
Allergic endotypes classifications	Characterization of different allergic endotypes for personalized therapies	Adel-Paxtient et al. [69] 2019
Pathogenetic mechanisms insight	Clinical and environmental factors associated with SU acquisition during BF or therapy (enriched FM, OIT) Evaluation of persistence of allergy or transient desensitization or SU	Berni-Canani et al., 2015 [67] Seppo et al. [68] 2017 Shibata et al. [70] 2023 Boulangé et al. [71] 2023

Abbreviation: FM, formula milk; OIT, oral immunotherapy; BF, breastfeeding; SU, sustained unresponsiveness; DBPCFC, double-blind, placebo-controlled food challenge; OFC, oral food challenge.

## Data Availability

Not applicable.
